# Hemostatic abnormalities after trauma resuscitation: challenges and strategies in caring for the critically injured patient

**DOI:** 10.1186/s13613-025-01587-0

**Published:** 2025-10-18

**Authors:** Christopher R. Reed, Nicola Curry, Nicole P. Juffermans, Matthew D. Neal

**Affiliations:** 1 Division of Trauma, Acute, and Critical Care Surgery, Department of Surgery, Duke University School of Medicine, Box 2837 DUMC, Durham, NC 27710 USA; 2 Medical Sciences Division, Radcliffe Department of Medicine, University of Oxford, Oxford Haemophilia and Thrombosis Centre, Nuffield Orthopaedic Centre, Oxford, OX3 7LD UK; 3 Department of Intensive Care Medicine, Laboratory of Translational Intensive Care, Erasmus Medical Center, Dr. Molewaterplein 40, Rotterdam, 3015 GD Netherlands; 4 Trauma and Transfusion Medicine Research Center, Department of Surgery, University of Pittsburgh, Keystone Building, 3520 Fifth Avenue, Suite 500, Pittsburgh, PA 15213 USA

**Keywords:** Trauma, Thrombosis, Thromboprophylaxis, Hemostasis, Injury, Coagulation, Coagulopathy, Hypercoagulable state, Acquired thrombophilia

## Abstract

Severe polytrauma and hemorrhage is a common and life-threatening condition often leading to intensive care unit admission for those who survive their initial injury. The injury itself, hypoperfusion from hemorrhagic shock, and resuscitative efforts introduce a complex set of hemostatic derangements collectively referred to as trauma-induced coagulopathy (TIC). Although the trauma population is notoriously heterogenous, TIC can generally be divided into an “early” hypocoagulable phase and then a “late” hypercoagulable, prothrombotic phase. Existing literature on TIC focuses heavily on reversing and preventing hypocoagulation in the early, acute phase. However, intensivists commonly manage patients throughout the later post-acute resuscitation phase of TIC, during which thrombotic complications are common and may lead to major morbidity and mortality. Derangements in platelet activation, endothelial dysfunction, suppression of fibrinolysis, and crosstalk between the innate immune and coagulation systems all contribute to the prothrombotic late TIC phenotype. Deep venous thrombosis and other macrovascular thrombotic complications also commonly occur after trauma. Thrombosis prophylaxis and treatment present a challenge for patients still at high risk for bleeding. An in-depth understanding of risk factors specific to trauma patients, including iatrogenic contributions from resuscitation and hemostatic efforts in the pre-intensive care phase, can help stratify thromboembolic risk and optimize prophylaxis and surveillance efforts. We stress the importance of an individualized approach to assessment of hemorrhagic and thrombotic risks for each patient. Here, we summarize the underlying contributors to the prothrombotic phenotype in late TIC, including a description of emerging roles for HMGB1, extracellular vesicles, and endogenous inhibitors. Additionally, a general approach to thromboprophylaxis, monitoring, and anticoagulation in this patient population are discussed. Finally, we summarize relevant risk stratification systems and guidelines for clinical management of thromboembolic risk among trauma patients, and highlight limitations in these systems and guidelines as areas for future research.

## Introduction to hemostatic derangements after trauma

Trauma induces a complex set of hemostatic derangements that have been the subject of considerable study over the past three decades [1]. Much of the effort to better understand and treat deleterious changes in coagulation and endogenous hemostasis has focused on the hyperacute phase of evaluation and resuscitation before definitive hemostasis is achieved, typically in the emergency department immediately after arrival or even prior to hospital arrival. This “early” trauma-induced coagulopathy (TIC) is characterized by a hypocoagulable state with a tendency toward hemorrhage, typically within 6 h of the initial injury. Factors related to the injury itself, subsequent resuscitation, and shock state from tissue hypoperfusion in the setting of hemorrhage all contribute to early TIC [2]. Many early preventable deaths after traumatic injury are effected by exsanguination, and increasing time between injury and hemostasis is associated with mortality [3, 4]. Therefore, measures to maximize coagulation potential and augment endogenous coagulation are employed in this hyperacute phase of resuscitation.

Much less attention has been given to hemostatic complications that can occur in the later phase of trauma care, at which point the patient is typically admitted to an intensive care unit (ICU). “Late” TIC is often defined as occurring >24 h after injury, although phenotypes can differ considerably within this time period. Traumatic injury is a major source of death and disability that increasingly affects patients across the spectra of age, medical complexity, socioeconomic status, and other demographics [5]. Severe injury is a common reason for admission to the ICU at trauma centers. In one study, half of all trauma patients admitted to Level 1 or Level 2 trauma centers were admitted to an ICU at some point during hospitalization, with a third of patients admitted directly to the ICU after emergency department evaluation [6].

Following this overview of TIC pathogenesis, the present review will aim to describe the current mechanistic understanding of late TIC and associated thrombotic risk. First, it will provide an overview of “late” TIC and introduce the timeline for hemostatic derangements after injury. Next, it will review contributions from the deranged soluble cellular, endothelial, and soluble acellular components of hemostasis. Next, it will provide an overview of clinical considerations facing the intensivist managing patients in this setting. Finally, it will provide a summary of current recommendations that address common scenarios and emerging questions regarding thromboprophylaxis among critically injured patients.

## Overview of late trauma-induced coagulopathy

**Fig. 1:**
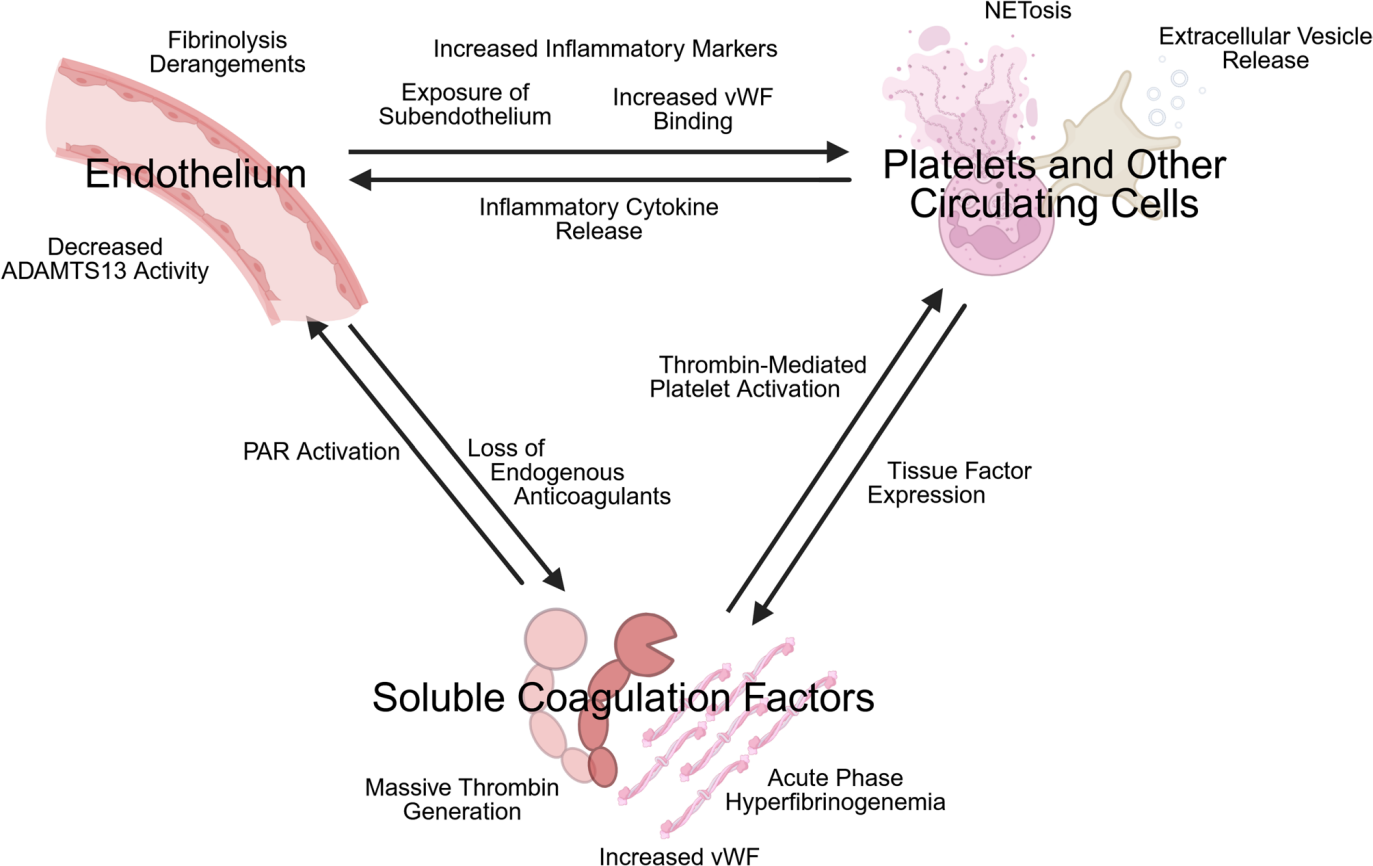
Visual schematic of major contributors and interactions leading to the subacute hypercoagulable state following traumatic injury. The major contributors to the overall prothrombotic state after injury can be summarized as the endothelium and its components, platelets and other circulating cells, and soluble plasma coagulation factors. However, these systems are highly connected and dependent on one another, as represented schematically by arrows. vWF: von Willebrand Factor. PAR: Protease-Activated Receptors

The response to shock and factors related to the injury itself contribute to hemostatic derangements leading to the “late” TIC hypercoagulable phenotype. Although some have suggested that resuscitation efforts may also be associated with late hypercoagulable complications, it is challenging (if not impossible) to separate this from the survivor bias of successfully resuscitated patients living long enough to experience thrombotic events [7–10]. After initial resuscitation, trauma patients become increasingly heterogenous, and a nuanced examination of coagulation potential in these patients will yield differing and even competing tendencies toward clotting and bleeding. This heterogeneity demands individualized monitoring and treatment for patients in this population. For example, fibrinolysis shutdown may contribute to thrombosis even in the setting of fibrinogen consumption with bleeding [11]. In fact, post-acute hemostatic derangements are currently an active area of study, with several different phenotypes emerging [12]. The variability of these phenotypes and their complex temporal relationships with injury, shock, and resuscitation reflect the complexity of the endogenous hemostatic system and its components (Fig. 1). For the purposes of this review, patients in the post-acute or late phase of TIC will generally be described as overall pro-thrombotic but still at risk for bleeding.

What is clear is that these complex derangements are responsible for considerable morbidity in the ICU setting following definitive hemorrhage control. Some of the most familiar and feared complications in this population can be attributed to the prothrombotic state rendered by trauma; for example, venous thromboembolism. Venous thromboembolism (VTE) includes deep venous thrombosis (DVT), pulmonary embolism (PE), portomesenteric venous thrombosis, and cerebral sinus thrombosis; although any venous distribution can certainly be involved [13]. Polytrauma is a major independent risk factor for inpatient VTE [14, 15]. Although injury itself is an independent risk factor for VTE, many other associated conditions common to trauma patients (immobility, spinal cord injury, recent surgery, central venous access) also independently contribute. These additional familiar states of thrombophilia often co-exist in the critically ill polytrauma patient and conspire to promote thrombosis at multiple sites in both native and extracorporeal (i.e., renal replacement system, central venous catheter) circulatory sites.

## Mechanisms of subacute prothrombotic state after polytrauma

### Platelet activation

Platelets play a central role both in maintaining normal hemostasis under unstressed, homeostatic conditions, as well as in reestablishing hemostasis after injury. The myriad adaptive mechanisms by which they contribute have been previously reviewed; their complexity and range of contributions belies their simple structure [16, 17]. These contributions include crucial mechanical, immunologic, biochemical, and hormonal roles; which differ in the immediate (24 h) and late TIC periods.

As with other areas of research in the field, most investigation into platelet function after injury has focused on the immediate hypocoagulable phase of injury [18]. So-called platelet “exhaustion” in which hyporeactive platelets contribute poorly to hemostasis may be in response to overwhelming pro-inflammatory and thrombotic stimuli, typically becoming most pronounced within 24–48 h of injury [19]. However, after this initial period of tendency toward hemorrhage, platelets drive thrombosis risk through multiple mechanisms in addition to their “primary” and best-described function as a site of thrombin generation. By post-trauma day 3, platelets become the dominant contributor to clot strength, superseding the contribution of fibrin as measured by thromboelastography (TEG) [20]. This contribution may be refractory to conventional thromboprophylaxis with low molecular weight heparin.

Another means by which platelets contribute to late TIC is by the release of prothrombotic extracellular vesicles, as well as through interaction with tissue factor-laden vesicles released by inflammatory cells. Immediately after injury, there is a massive systemic release of extracellular vesicles [21, 22]. Although the specific role of these heterogeneous, submicrometer, anucleate bodies is still under study across the spectrum of human health and disease, they clearly serve procoagulant and proinflammatory functions and are associated with thrombosis [23, 24]. In fact, platelet-derived extracellular vesicles are so potently hemostatic that they have been proposed as a cell-free resuscitation medium in patients with hemorrhagic shock and ongoing bleeding [25, 26]. Their efficacy in promoting hemostasis in this setting unsurprisingly may come with a delayed risk of thrombosis. In an adoptive plasma transfer experiment using a rodent model of liver trauma and DVT, platelet-derived microvesicles effected both hemostasis and thrombosis in the form of diminished liver laceration bleeding and increased venous thrombosis volume, respectively [27]. It was also recently discovered that thrombin can mediate the release of additional extracellular vesicles from platelets, which then promotes thrombin activation on platelet surfaces, setting up a powerful feedback loop [28]. Interestingly, activated platelets may also promote extracellular vesicle-mediated thrombosis indirectly by way of P-selectin and PSGL-1 binding to tissue factor-laden vesicles from other inflammatory cells [29]. This interaction exposes phosphatidylserine, a key lipidic scaffold for coagulation factor activation [30]. Unfortunately, their diversity in content, size, and surface markers presents a challenge in developing specific therapeutics to modulate vesicle-derived prothrombotic tendency after trauma. Currently, studies are underway to phenotype circulating extracellular vesicles following injury, which may implicate specific signaling pathways or other pathologic mechanisms by which they effect thrombosis and yield targets for intervention [31, 32]. These studies represent the first steps toward exploiting platelet-derived microvesicles as biomarkers, hemostatic adjuncts, and effectors of thrombotic risk.

Platelets may also contribute to thrombotic complications by way of innate immune activation. Beyond their primary role in maintaining hemostasis, platelets have an important secondary function in promoting normal immunity. This positions platelets as a key mediator between the coagulation and innate immune systems [33]. The links between coagulation and inflammation have been the subject of increasing study and scrutiny over the past decade, with major ramifications for human health and disease [34]. In fact, the role of platelets in mediating or attenuating inflammatory disease severity and thromboinflammation has been well established [35]. As an example, the role of platelet surface high mobility group box 1 (HMGB1) protein has emerged as both a potential biomarker as well as target for intervention in multiple thrombotic disease states, including trauma [36]. Classically, HMGB1 is a damage-associated molecular pattern arising from multiple cell types that may circulate after tissue injury or infection, Inflammatory cells may release soluble HMGB1 or nuclear HMGB1 may be released after cell death. It may propagate inflammatory signaling through toll-like receptors 2 and 4 [37, 38]. This makes HMGB1 a crucial link between sterile and infectious inflammatory states; i.e., trauma and sepsis. However, platelet surface-specific HMGB1 has been implicated in both normal hemostasis and pathologic thrombosis after traumatic injury [39, 40]. Elevations in circulating HMGB1 can be detected immediately after injury, but supranormal levels persist for at least 5 days after injury, well into the prothrombotic ICU care phase of TIC [41]. Therapeutic inhibition of circulating HMGB1 is under preclinical investigation, although the feasibility and efficacy of a platelet-focused inhibition strategy has not yet been studied [42–45].

Through a process often referred to as immunothrombosis, platelets also help orchestrate the development and localization of neutrophil extracellular traps (NETs) that are another important link between innate immunity and thrombosis after injury. Although neutrophils are capable of NETosis and chromatin release without platelet contributions in vitro, activated platelets can effect nonlytic NETosis, an important innate immune pathway best studied in sepsis [46, 47]. This bidirectional activation yields pathologic intravascular thrombosis that causes organ dysfunction [48]. The role of NETosis in post-traumatic organ dysfunction, inflammation, thrombosis, and tissue healing is an active area of research, with most studies on the subject historically focusing on infectious conditions [49, 50].

### Endotheliopathy

The endothelium is a complex, dynamic, and large organ that physically interfaces with every other organ system in the body and generally maintains a fibrinolytic and anticoagulated phenotype under unstressed conditions [51]. As with platelets, it plays an important and complex role in maintaining and restoring normal hemostasis; these contributions have been previously reviewed [52]. Immediately following trauma, it protects against inappropriate intravascular thrombosis via its physical barrier between circulating blood and the prothrombotic subendothelium, a site rich in tissue factor and collagen [53, 54]. The luminal surface of the endothelium is coated with the glycocalyx, a layer of proteoglycans and glycoproteins responsible for physiochemical promotion of an anticoagulant surface by (a) physically separating circulating platelets and coagulation factors from the subendothelium, and (b) positioning endogenous anticoagulants (i.e., heparan sulfate) and fibrinolytics (i.e., tissue plasminogen activator [tPA] and urokinase plasminogen activator [uPA]) at the blood-endothelium interface [55, 56].

The initial pathogenesis of early endothelial injury after trauma, including glycocalyceal shedding, activation of endothelial cells, and increased adhesion of pro-inflammatory and thrombotic leukocytes and platelets, has been reviewed in detail previously [57]. Later hemostatic derangements arising from traumatic endotheliopathy can be conceptualized as those related to breakdown of the vascular barrier (i.e., glycocalyx shedding), and others related to loss of endogenous luminal anticoagulation and fibrinolytic molecules.

Endothelial damage contributes to organ dysfunction and thrombotic risk for at least one week after injury based on elevations of glycocalyx components in circulation through that time [58, 59]. During this timeframe, increased markers of endothelial damage are found in circulation, and increased soluble von Willebrand Factor (vWF) promotes platelet and leukocyte adhesion to the injured endothelium. This yields an overall prothrombotic tendency [60, 61]. ADAMTS13 level is decreased early in injury, resulting in increased circulation of ultra-large vWF multimers [62–65] and thrombi in organs. Mismatch between circulating vWF and ADAMTS13 in the first week after injury contributes to thrombotic tendency. A prospective study in trauma patients noted that ADAMTS13 activity tended to recover by post-injury day 6, suggesting that thrombotic risk imparted by this derangement may abate while the most critically injured patients remain in the hospital [66].

Therapeutic approaches to protecting or restoring the endothelium after trauma are largely focused on the role of blood products during initial resuscitation. There is substantial evidence that one or more endogenous plasma molecules may prevent endotheliopathy with early plasma-based resuscitation. Although a variety of candidate molecules have been identified, all are constituents of plasma and underscore the importance of using an early hemostatic resuscitation strategy that minimizes crystalloid and relies on whole blood or balanced transfusion of products [67–69]. It is also worthwhile to note that plasma transfusion may attenuate late prothrombotic tendency by restoring normal plasma anticoagulants and preventing endothelial injury. This may seem counterintuitive, since plasma transfusion is often utilized to treat bleeding tendency. Finally, preclinical investigations suggest a promising role for ADAMTS13 transfusion in trauma patients to reduce leukocyte and platelet adhesion that may contribute to intravascular thrombosis [63].

### Derangements in fibrinolysis

Like the plasma coagulation system, the normal fibrinolytic system is comprised of a complex network of soluble enzymatic proteins, cofactors, receptors, and inhibitors that have previously been reviewed elsewhere [70]. Its activity is subject to tight regulation by multiple interactions with other related elements of coagulation (platelets, thrombin, fibrinogen). Plasmin is the main effector enzyme of fibrinolysis. Its best-described activity is to dissolve fibrin-based clots, although it degrades many other proteins important for normal hemostasis, activates platelets, and aids in cell migration, immunity, and survival [71–73]. The fibrinolytic system’s polyfunctionality represents a primordial connection between immunity and thrombosis that long predates the coagulation system’s better appreciated crosstalk between inflammation and thrombosis in an evolutionary sense [74]. Plasminogen circulates as a zymogen and requires proteolytic activation by tPA to maximize fibrinolytic activity. There are multiple important circulating endogenous inhibitors of plasmin that work by diverse and often indirect mechanisms. The primary inhibitor of plasmin, alpha-2-antiplasmin, forms an irreversible covalent serpin-protease intermediate complex after binding to soluble plasmin in circulation [75, 76]. Conversely, plasminogen activator inhibitor-1 (PAI-1) targets plasminogen activators, indirectly inhibiting fibrinolysis and conferring implications on other biologic processes [77]. Finally, thrombin activatable fibrinolysis inhibitor (TAFI) is a soluble zymogen carboxypeptidase that, when activated by thrombin, exerts antifibrinolytic effects and is an example of coagulation-fibrinolysis crosstalk that promotes autoregulation of clot formation in vivo [78].

Efforts have been made over the past two decades to better describe a series of postinjury fibrinolytic phenotypes and their associations with injury severity, time after trauma, and clinical tendency to bleed or clot. These phenotypes are broadly termed hyperfibrinolysis, physiologic fibrinolysis, and fibrinolysis shutdown [79]. A recent and elegant rodent study of tPA, alpha-2-antiplasmin, and PAI-1 levels after severe blunt trauma concluded with a narrative description of a common scenario in the trauma ICU: “Immediately after trauma, the fibrinolytic system was activated; however, its activation was quickly and intensely suppressed [80]”. In rats, this conversion from hyperfibrinolysis to fibrinolysis shutdown occurred biochemically over the course of minutes to hours. In clinical studies of human trauma patients utilizing TEG or PAI-1 as a surrogate for fibrinolysis shutdown, signatures of shutdown may be detected between 1 and 12 h after injury [12, 81, 82]. Shutdown is a common phenotype after moderate or severe injury. In a large study of trauma patients with an injury severity score (ISS) of at least 15, there was a fibrinolytic shutdown rate on admission TEG approaching 50% [12]. Risk factors include older age and TBI. The association between shutdown and poor outcomes, including mortality, has been established [12, 82–85].

A study by Meizoso and colleagues found that 44% of patients admitted to an ICU with a fibrinolytic shutdown phenotype via TEG on admission had persistent shutdown a week after their injury, highlighting the ongoing challenges posed to intensivists managing severely injured patients beyond their acute presentation. It is worthwhile to note that ROTEM and TEG are technically limited in their abilities to quantify fibrinolysis. Their ubiquitous use in clinical practice makes them convenient but not very specific tools with which to compare patients. Further studies utilizing D-dimer or plasmin-plasmin inhibitor complexes might provide more specific interrogation of fibrinolysis in future studies. Exactly how fibrinolysis shutdown could influence decisions regarding thromboprophylaxis remains to be seen.

### Massive tissue factor release and thrombin generation

Tissue factor is an integral membrane protein that is expressed in the vascular adventitia, capsules of organs, and other sites out of direct contact with blood. In the modern cell-based view of hemostasis, clotting is typically initiated by a vascular injury or other prothrombotic stimulus. This results in exposure of a tissue factor-bearing cell to the blood’s enzymatic coagulation factors and cofactors, with complexing to Factor VIIa initiating the process of forming insoluble clot [86]. Under normal homeostatic conditions, tissue factor is only expressed in cells comprising a “hemostatic envelope” (i.e., fibroblasts of the adventitia, cells comprising the capsule of solid organs, and cells of the epidermis, bowel, and respiratory mucosae). However, stimulation of monocytes and dendritic cells with proinflammatory cytokines, such as those encountered after trauma and in critical illness, induces expression of tissue factor that may contribute to thrombosis [87, 88]. Appearance of inducible tissue factor occurs shortly after injury owing to exposure of leukocytes to tumor necrosis factor alpha (TNF-α), IL-1β, and IL-6; these cytokines may have protracted elevations days or even weeks into hospital admission [89–91]. Massive tissue factor release in the setting of ongoing inflammation may lead to uncontrolled and inappropriate thrombin generation, culminating in microvascular thrombosis. Thrombin generation is greater in trauma patients than in healthy patients, independent of shock [92, 93]. Unsurprisingly, thrombin generation in this patient population has also been associated with development of thromboembolic events within 30 days of injury, despite levels decreasing within the first day of admission [94].

### Additional and emerging prothrombotic influences after trauma

Although platelets, the endothelium, fibrinolysis, and soluble plasma coagulation factors play central roles in the subacute prothrombotic phenotype following trauma, additional contributing factors are emerging that might yield exciting targets for treatment and thromboprophylaxis.

One such emerging factor is skeletal muscle myosin, a large motor protein involved in sarcomere contraction. In a recent exome genotyping study of 107 patients with VTE, a single nucleotide polymorphism in the skeletal muscle myosin heavy chain was implicated in patients with recurrent VTE [95]. The same study demonstrated that skeletal muscle and cardiac muscle myosin are capable of replacing lipid and membrane contributions from platelets and organizing the prothrombinase complex necessary for thrombin generation independently [96]. Additionally, soluble skeletal muscle myosin has correlated with traditional markers of coagulation status after trauma; yielding the finding of low circulating myosin correlating with hypocoagulability [97]. This has obvious implications for thrombin generation and thrombosis after traumatic injury which may release soluble myosin into plasma. Anti-myosin antibodies were able to blunt thrombin generation with even greater efficacy in plasma from trauma patients compared to controls [98]. Skeletal muscle myosin in trauma may be a biomarker for thrombotic risk or target for prophylaxis or therapy in the future.

Inorganic polyphosphates (i.e., “polyP”) form a class of thrombogenic molecules found in platelet granules and mast cells. They are also synthesized by some bacteria and may play a role in evasion of host immune responses. Classically, the function of human polyP had been limited to activation of Factor XII to XIIa, and was therefore not considered to be critical in normal hemostasis. However, more recent focus on the link between thrombosis and inflammation has yielded additional insight into the thrombogenic potential of polyP, which is presumably released after platelet and mast cell degranulation following trauma [99]. A recent study quantified thrombin generation from trauma patients and controls before and after exposure to polyphosphate inhibitors and demonstrated a reduction in thrombin generation among trauma patient samples [100]. The authors conclude that polyP (or another related polyanion) contributes to thrombin generation and thrombosis after injury. Such inhibitors may help attenuate this response and are in preclinical testing currently [101].

Although further research is needed to delineate the roles of these molecules in the prothrombotic state after trauma, both are testaments to how our understanding of traumatic coagulopathy continues to rapidly evolve.

Although impressive advances in our understanding of late TIC and the associated hypercoagulable state have yielded these mechanistic insights, optimal management of late TIC remains an important knowledge gap. Ongoing questions about the role of heparinoids to prevent or treat microvascular thrombosis, plasma-based resuscitation protocols for hemorrhagic shock, and cold-stored or synthetic platelet products are all emerging therapies aimed to mitigate effects of injury and shock on hemostasis.

## Clinical management of late trauma-induced hypercoagulability

### Endogenous risk factors for thrombosis

Venous thromboembolism is a common and potentially preventable cause of significant morbidity and mortality in trauma patients [102]. As many as 1 in 5 of those who develop PE after injury will die [103, 104]. Studies reporting the incidence of injury-associated VTE vary widely in their estimates from 7 to 58%, with the highest rates in cohorts where pharmacological thromboprophylaxis is not offered, and where VTE is detected by unselected screening in all patients regardless of symptoms [105, 106]. Even higher rates have been reported in military populations compared to civilians [107]. Recent data also support that many pulmonary arterial clots discovered early after injury may not be embolic but may be local events in the lung resulting in pulmonary thrombosis in situ [108]. Whether these events should be treated in the same way as pulmonary emboli is unclear, and these events are not preventable due to their recognition immediately after injury.

### Exogenous risk factors for thrombosis

Some therapies directed at treating TIC during bleeding may increase the risk for VTE. When given empirically to bleeding trauma patients, prothrombin complex concentrate was associated with higher VTE rates due to excess thrombin generation [109, 110]. The impact of tranexamic acid (TXA) on VTE has been highly debated, as some studies and initial meta-analyses have demonstrated an increased VTE risk, but others have not [111, 112]. One such analysis concluded that results from the largest studies of TXA and thrombosis risk should not be pooled, making it even more challenging to discern overall risk [111]. The latest meta-analysis incorporating a large trial from 2023 again suggests an increased risk of VTE with the use of TXA, with an odds ratio of 1.22 and confidence interval of 1.03–1.44 [112, 113]. Ultimately, none of the largest trials have confirmed TXA administration to be an independent risk factor for VTE after traumatic injury.

Neither administration of fibrinogen nor specific blood product transfusion ratio appear to be associated with VTE [114]. Surgery following trauma is a clear risk factor for VTE with an adjusted odds ratio of 2.60 and 95% confidence interval of 1.66–4.07 [115]. Delays in pharmacologic VTE prophylaxis greater than 24 h are associated with an approximately two-fold increase in VTE risk, and missed doses of prophylaxis are also a common factor associated with VTE. Conversely, use of anticoagulants prior to injury is associated with reduced risk of VTE [115]. Selected known risk factors for VTE are listed in Table 1.

**Table 1:** Risk factors for VTE after trauma

Endogenous	Exogenous
*Patient-related*	*Empiric treatment*
Advanced age	PCC
Obesity	Tranexamic acid?
Male sex	Factor VIIa
*Injury-related*	*Management*
Injury severity	Surgery for trauma
Pelvic fracture	Delayed pharmacologic prophylaxis
Lower extremity fracture	
Spinal injury	
Penetrating injury	

### Prediction scores for venous thromboembolism

The Padua prediction score is a well-validated score for VTE risk in non-surgical hospitalized patients, in which the presence of trauma is also considered. In addition, scores specific to the trauma population have been developed. The risk assessment profile (RAP) score is a numeric risk score calculated within 24 h of admission that considers age, specific patterns of injury, iatrogenic factors (such as blood transfusion or femoral central venous catheter), and underlying medical conditions that contribute to thrombophilia [116]. This model has been validated in multiple studies, but its limitations (including complexity of calculation) have also been well established [117]. The Trauma Embolic Scoring System (TESS) score was developed using 17,000 patients from a U.S. trauma center to develop a simpler risk assessment model. The TESS model considers age, ISS, obesity, mechanical ventilation, and lower-extremity trauma, as these were significant predictors of VTE. A TESS score of more than 6 had a sensitivity of 82% to predict VTE, with an 84% specificity. Overall, the model had excellent discrimination in predicting VTE with a receiver operating characteristic curve of 0.89 [118]. In another study using patients from a single trauma center in China, a score to detect DVT was developed with similar variables, including age, ISS, body mass index, lower extremity fracture. These clinical variables were augmented with coagulation tests (D-dimer, fibrin degradation products, and prothrombin time), yielding the DVT Risk Assessment Score (DRAS). This DRAS predicted DVT with an area under the receiver operating characteristic curve of 0.89 [119]. Of note, pulmonary thrombosis is not addressed by this score. Notably, both cohorts were derived from single centers and external performance of these models is uncertain. Additionally, most patients in the sample used to create the TESS scoring model were not critically injured, and less than 10% of these patients required mechanical ventilation, limiting its ability to discern risk in an ICU setting. Measures of critical illness (i.e., mechanical ventilation and ICU admission) are not reported for the DRAS cohort. This highlights the need to better understand important predictor variables of VTE following trauma and whether these variables are widely applicable.

### Ultrasound screening

Routine screening with ultrasound increases the incidence of VTE to 23% as compared to 10% incidence when imaging is performed on clinical suspicion [114]. This suggests a substantial group of patients with minimally or asymptomatic VTE. It is possible that routine screening may result in unnecessary start of anticoagulation with subsequent bleeding risk despite no benefit. However, routine screening may also prevent the development of major pulmonary embolism or other complications of DVT. The risk-benefit of this question was addressed in a single center trial in which trauma patients with a median ISS of 14 were randomized to routine lower extremity DVT screening with ultrasonography on days 1, 3, 7, and then weekly vs. usual care [120]. Routine screening indeed resulted in fewer in-hospital PEs, albeit without an effect on mortality. There were no differences in anticoagulant-associated bleeding between the arms. Notably, DVT often occurred within the first days after trauma, often even before initiation of pharmacologic thromboprophylaxis. In situ pulmonary thrombosis may even occur at the time of injury. These findings are in accordance with a single-center retrospective study showing lower rates of PE in patients who were routinely screened for DVT [121]. Therefore, routine screening with ultrasonography may have a place following severe trauma among patients at greatest risk of PE.

### Viscoelastic hemostatic testing

Viscoelastic hemostatic testing includes rotational thromboelastometry (ROTEM) and thromboelastography (TEG). The role of these tests in monitoring and treating bleeding tendency is well established, but these tests can also detect hypercoagulability. Conventional coagulation and hematologic tests have little or no predictive value in estimating thrombotic risk for critically ill patients, and fibrinogen degradation products are also less informative in post-trauma and post-operative patients. Most associated work has been done in the surgical patient population. A meta-analysis of studies in post-surgical patients demonstrated that a maximum amplitude (MA) of 65 mm on the first day after surgery for trauma was associated with VTE risk, albeit with a low predictive ability [122]. A study in patients requiring surgery following traumatic hip fracture showed that a clot formation time (CFT) of less than 65 s preceding surgery had an excellent performance to predict the occurrence of symptomatic VTE after surgery [123]. With respect to trauma patients not undergoing surgery, a single-center prospective study showed that a hypercoagulable viscoelastic test (defined as reaction time (R) below, angle (α) above, or MA above reference ranges) is associated with increased risk of DVT in trauma patients, as assessed by routine screening with ultrasound of extremities [124]. Of note, 85% of trauma patients in this study were hypercoagulable on admission, which is a remarkably high rate, when compared to 7% as found in another study in which hypercoagulability was defined as increased G value [125]. The frequency of hypercoagulability in this patient population that may be actively bleeding and that remains at risk for hemorrhage nicely illustrates the dilemma faced by intensivists. Both ROTEM and TEG may help clinicians stratify the risk of thrombosis and hemorrhage in individual patients, where these tendencies may be heterogenous. Taken together, viscoelastic hemostatic testing shows promise to prognosticate VTE after trauma but the multiple definitions of hypercoagulability and current sparsity of data hamper any clear recommendations presently.

### Thromboprophylaxis in the polytrauma ICU patient

Robust thromboprophylaxis strategies have been shown to reduce VTE rates by at least 3-fold [102, 104, 126]. Chemical thromboprophylaxis is now universally accepted as the standard of care for hospital inpatients [127–130]. However, variations in clinical practice remain, partly driven by the differences in recommendations among international guidelines (Table 2), but also due to uncertainty about how to optimize outcomes for individual patients with competing thrombotic and bleeding risks. This balance of risk is exemplified by the polytrauma patient. Not only do these patients usually have the highest risk for VTE among all inpatients while they may still be bleeding or require procedures; but they are also more likely to develop organ failure, e.g. acute renal dysfunction with attendant bleeding risks.

**Table 2:** Summary of thromboprophylaxis guidelines from relevant professional societies

	AAST Guidelines - Rappold 2021 (ICU) - Yorkgitis 2022 (Inpatient) - Berndtson 2024 (Discharge)	ASH Guidelines - Anderson, 2019	European - C Heim 2024, Rossaint, 2023 (Pan-European Task Force) - Heim 2023 (European Society of Anaesthesiology and Intensive Care)	NICE - NG89, 2019
Type of guideline	Trauma specific	General	Trauma specific	General
*Mechanical Thromboprophylaxis*				
Graduated Compression Stockings	Not specified	Recommends use of mechanical TP (unspecified), particularly where pTP cannot be used	Recommends against their use	Can be used as a mechanical TP method
Intermittent Pneumatic Compression	Not specified	Recommends use of mechanical TP (unspecified), particularly where pTP cannot be used	Recommends using IPC devices in all, and particularly in patients where the bleeding risk prevents pTP	Offer to all patients with major injury and continue until mobility is no longer significantly reduced
Inferior Vena Cava Filters	Does not support routine placement. Only to be used for very high-risk individuals who cannot receive pTP for extended periods	Recommends against IVC filter use	Recommends against IVC filter use	Suggests not to use IVC filters routinely
*Pharmacological Thromboprophylaxis (pTP)*				
Timing of pTP	Early use of pTP – Guided by a scoring system Low bleeding risk: Immediate use Moderate to higher bleeding risk: Within 48 h	Low to moderate bleeding risk: use pTP High bleeding risk: do not use pTP	Start pTP < = 24 h after control of bleeding	Consider pTP as soon as the thrombotic risk outweighs the bleeding risk Assess daily
Drug recommended	LMWH (Enoxaparin)	LMWH or UFH	LMWH	LMWH
Dose of pTP	30 mg b.i.d. – if > 65 y, < 50 kg, CRTCL < 60 ml/min If < 65 y, > 50 kg, CRTCL > 60 ml/min: 40 mg b.i.d. if BMI also < 30 0.5 mg/kg b.i.d. if BMI > 30	Not specified	Dose adjustment preferred (but insufficient evidence to support one method)	Not specified
Weight adjusted	Yes – as above (weight and/or BMI adjusted)	Not specified	Yes (or anti-Xa)	Not specified
*Coagulation monitoring to guide pTP:*				
Anti-Xa	Aim: peak – 0.2–0.4 iu/ml Trough 0.1–0.2 iu/ml	Not specified	Insufficient evidence	Not specified
Thromboelastography	Not specified	Not specified	No	Not specified
LMWH or UFH in renal dysfunction	Defined as a CrCl < 30 ml Use 5000 IU UFH t.i.d. (BMI < 30) 7500 IU UFH t.i.d. (BMI > 30)	Not specified	Not specified	Not specified
Duration of pTP	For in-patient stay	Not specified	Not specified	pTP for a minimum of 7 days, and during in-patient stay
DVT surveillance	Scan asymptomatic patients only when they are at very high risk for VTE	Not specified	Not specified	Not recommended
Extended pTP beyond in-patient stay	Extremity or pelvic fracture − 28 days aspirin 81 mg b.i.d. Spinal cord injury – 3 months LMWH TBI – 28 days LMWH (LMWH = enoxaparin 30 mg b.i.d.)	Not specified	Not specified	No specific recommendation for extended pTP beyond 7 days for trauma patients

*AAST* American association for the surgery of Trauma,* ASH* American society of Hematology,* b.i.d.* twice daily,* BMI* body mass index,* crcl* creatinine clearance,* IU* international units,* IVC* inferior Vena cava,* LMWH* low molecular weight heparin,* mL* milliliter,* NICE* National Institute of health and care Excellence,* TBI* traumatic brain injury,* t.i.d* three times daily,* TP* thromboprophylaxis,* UFH*: unfractionated heparin,* VTE* venous thromboembolism,* xa* activated factor X

### Type of thromboprophylaxis

#### Mechanical

Graduated compression stockings and intermittent pneumatic compression devices (IPC) are commonly used methods for reducing hospital-associated VTE. The efficacy of compression stockings in the trauma setting has not been tested in a randomized controlled trial, but there has been a general move away from their use following the results of the GAPS trial that failed to show benefit across all hospitalized patient groups [131]. In contrast, higher quality data support the use of IPC devices. A recent Cochrane review concluded that the combination of IPC and pharmacological thromboprophylaxis, when compared with either strategy as a single agent, reduced the risk of both PE and DVT [132]. Although this benefit came at the expense of higher rates of clinically important bleeding, this risk was not seen with IPC devices alone.

#### Inferior vena cava (IVC) filters

Most commonly, IVC filters are inserted prophylactically or as part of a treatment strategy in patients who cannot receive anticoagulation due to unacceptable bleeding risk. A recent meta-analysis concluded that prophylactic IVC filters may confer a moderate risk reduction in PE, but increased DVT risk leads to a lack of consensus that IVC filters are beneficial [126]. Guidelines suggest against routine placement of IVC filters in patients able to be anticoagulated or for primary prophylaxis [133]. In situations where they are used, there is also universal agreement that IVC filters should be removed as soon as is clinically possible. This strategy is supported by RCT data from Australia, where no long-term sequelae were reported in 198 patients where the median duration of IVC filter placement was 55 days [134].

### Pharmacological thromboprophylaxis

Guidelines from US and European professional societies recommend low molecular weight heparin (LMWH) as first-line pharmacological thromboprophylaxis, with unfractionated heparin as an alternative, particularly for those with significant renal dysfunction [127, 135]. Recommendations on the use of fixed doses (often graded according to weight-based ranges), or in some cases guided by anti-Xa measurements, vary considerably. Importantly, patients with TBI are subject to additional considerations given their risk for intracranial hematoma expansion and are not addressed here. Given the convenience of oral administration, there has also been increasing interest in aspirin and direct oral anticoagulants (DOACs) for prophylaxis as well. Aspirin monotherapy for prophylaxis is discussed later in this review. Although existing studies in polytrauma patients are retrospective, there is accumulating evidence that DOACs may be safe and effective for VTE prophylaxis after injury among patients with enteral access and no other contraindication [136–138].

#### Timing

Most international guidelines recommend starting pharmacological thromboprophylaxis early after injury, although specific timings vary from 24 to 48 h (American Association for the Surgery of Trauma and the European Society of Anaesthesiology and Intensive Care) to a more individualized approach of ‘when the thrombotic risk outweighs the bleeding risk’ (European and United Kingdom guidance). No randomized controlled trials have been performed to inform clinicians in this space. A large observational study of more than 14,000 individuals with severe trauma (ISS ≥ 15) reported that pharmacologic thromboprophylaxis within 24 h of admission was associated with the lowest incidence of VTE (3.0%) when compared to delayed treatment, where incremental increases in VTE rate occurred with increasing delay, as high as 9% if thromboprophylaxis was started after 72 h [139]. Similar results came from a prospective study in young adults, confirming a three-fold increase in VTE among patients in receipt of pharmacologic thromboprophylaxis after 24 h [140]. Importantly, although early prophylaxis is recommended, the observation of very early VTE (within 24 h) and in situ pulmonary thrombosis on admission suggest that thromboembolic events in trauma patients cannot and should not be considered “never events,” as there may be no opportunity to adequately prevent these early events [7, 108, 141].

*Weight-adjusted LMWH*: Data to support application of weight-based dosing of LMWH in trauma patients are limited. There are no randomized controlled trials in this space, but despite this, weight-based approaches are increasingly used. Secondary analysis of the CLOTT-1 observational study compared weight-based enoxaparin (0.45–0.55 mg/kg twice daily) with fixed doses (either 30 mg twice daily or 40 mg daily) in young patients (age 18–40 years). No difference in incidence of VTE was found between groups, adjusted odds ratio 0.75, 95% CI: 0.38–1.48. However, both strategies were found to be safe, with a 1.0% rate of LMWH-associated complications and no inter-group differences [140]. Of note, recent data report that antithrombin deficiency is common after severe injury, and this may contribute to resistance to heparinoids in trauma patients [142].

#### Monitoring anti-Xa activity

Monitoring of anti-Xa activity for prophylactic dosing of LMWH is not the standard of care in most countries or practiced at most institutions. Laboratory testing of anti-Xa activity during routine prophylactic administration of LMWH is typically reserved for excluding overdose where it may be clinically suspected. Observational data have reported that trauma patients will commonly have anti-Xa levels that fall below those expected, but data do not confirm that dose adjustments reduce rates of VTE. One explanation for this finding may be the delay in reaching prophylactic anti-Xa levels with dose optimization. Notably, neither of the two studies on the subject reported their choice of anti-Xa reagent, or whether this contained exogenous antithrombin [143, 144].

#### TEG guidance of LMWH dosing

A 2016 randomized controlled trial in 185 trauma and surgical patients compared a standard prophylactic regimen with 30 mg enoxaparin twice daily with TEG 5000-guided doses using standard citrate and heparinase samples. In the TEG-guided group, enoxaparin doses were titrated until standard TEG R-times were 1–2 min longer than TEG heparinase from the same specimen [145]. The authors found no difference in VTE, but there was an increase in bleeding (5.6% vs. 13.0%, *p* = 0.08), in the TEG-guided group, particularly in those patients in receipt of the highest enoxaparin doses.

#### Renal dysfunction

Despite enoxaparin’s renal clearance and the demonstrable increase in circulating drug concentration among patients with severe renal impairment, there may still be a role for prophylactic dosing of enoxaparin in this patient population. In the PROTECT trial, a pre-specified subgroup analysis of those with severe renal dysfunction at the time of ICU admission (creatinine clearance < 30 mL/min), including those with end-stage renal failure, showed no differences in bleeding or thrombosis rates when comparing dalteparin 5,000 IU daily with unfractionated heparin 5,000 IU twice daily [146]. Unlike with prophylactic dosing, there is increased bleeding risk among patients who are therapeutically (i.e., daily dosing with 1 mg/kg) anticoagulated with enoxaparin [147]. Low molecular weight heparin must be used with caution in patients with renal insufficiency.

*Aspirin for thromboprophylaxis*: Although LMWH is generally still accepted as the first-line choice for prophylaxis, there is some interest in the use of aspirin after considerable study in orthopedic surgical patients [148]. The PREVENT CLOT trial included 12,211 adult trauma patients with either an extremity fracture that required operation or a pelvic fracture. Patients were randomized to either enoxaparin 30 mg twice daily (standard prophylaxis) or aspirin 81 mg twice daily throughout hospital admission, and potentially after discharge according to local protocols [149]. Death was chosen as the primary endpoint. Mortality was 0.73% with LMWH, vs. 0.78% with aspirin (p = not significant) confirming noninferiority of aspirin with respect to mortality. Notably, DVT rates were higher with aspirin: 1.7% LMWH vs. 2.5%. This finding has been reproduced in additional large orthopedic LMWH vs. aspirin randomized controlled trials and which should give pause to clinicians on the universal adoption of aspirin monotherapy for thromboprophylaxis [150]. More research is needed to determine if the safety and efficacy of aspirin monotherapy is adequate in polytrauma patients.

A much smaller study (*n* = 329), the ADAPT single-center open-label trial, aimed to evaluate these drugs more ‘globally’ by using a composite endpoint of bleeding, wound infection, DVT, PE and death at 90 days. The same patient eligibility criteria were chosen, and twice-daily aspirin 81 mg was compared to LMWH (30 mg enoxaparin twice daily with anti-Xa adjustment). They found that LMWH offered between ‘no benefit’ to ‘moderate global benefit’ over aspirin, with a suggestion of greater event-free analysis with LMWH up to 90 days [151].

The addition of aspirin to LMWH has also been investigated. In a large (*n* = 10,532) retrospective analysis of a trauma database in the United States, the concomitant use of these two drugs led to a 43% reduction in relative VTE hazard [152].

## Conclusions

There is a rapidly accruing body of evidence examining the foundations of hemostatic dysfunction in the ICU after traumatic injury. These patients remain at high risk for thromboembolic and bleeding complications simultaneously, as well as thrombosis-mediated organ dysfunction. While exciting novel strategies aiming to treat this dysfunction are in the preclinical testing phase, early and aggressive guideline-driven thromboprophylaxis is paramount to minimizing morbidity associated with late TIC. Intensivists must be facile with thrombotic risk factors and prevention strategies in this patient population to provide optimum care.

## Data Availability

Not applicable.

## Abbreviations

TIC: Trauma-induced coagulopathy
VTE: Venous thromboembolism
DVT: Deep venous thrombosis
PE: Pulmonary embolism
TBI: Traumatic brain injury
TEG: Thromboelastography
HMGB-1: High mobility group box 1
NET: Neutrophil extracellular trap
tPA: Tissue plasminogen activator
uPA: Urokinase plasminogen activator
vWF: Von Willebrand Factor
PAI-1: Plasminogen activator inhibitor-1
TAFI: Thrombin activatable fibrinolysis inhibitor
ISS: Injury severity score
TNF-α: Tumor necrosis factor-alpha
TXA: Tranexamic acid
TESS: Trauma embolic scoring system
DRAS: DVT risk assessment score
ROTEM: Rotational thromboelastometry
MA: Maximum amplitude
CFT: Clot formation time
R-time: Reaction time
ICP: Intermittent pneumatic compression device
IVC: Inferior vena cava
LMWH: Low-molecular weight heparin
